# Geographical distribution of hyperuricemia in mainland China: a comprehensive systematic review and meta-analysis

**DOI:** 10.1186/s41256-020-00178-9

**Published:** 2020-11-30

**Authors:** Jiayun Huang, Zheng Feei Ma, Yutong Zhang, Zhongxiao Wan, Yeshan Li, Hang Zhou, Anna Chu, Yeong Yeh Lee

**Affiliations:** 1 Department of Health and Environmental Sciences, Xi’an Jiaotong-Liverpool University, Suzhou, 215123 Jiangsu China; 2 School of Medical Sciences, Universiti Sains Malaysia, 16150 Kota Bharu, Kelantan Malaysia; 3 Jinzhou Medical University, Jinzhou, 121001 Liaoning China; 4 Department of Nutrition and Food Hygiene, School of Public Health, Soochow University, 199 Ren’ai Road, Suzhou, 215123 China; 5 Department of Respiratory and Critical Care Medicine, The Second People’s Hospital of Wuhu, Wuhu, 241000 Anhui China; 6 Clinical Medical College, Yangzhou University, Yangzhou, 225009 Jiangsu China; 7 Department of Clinical Nutrition, Northern Jiangsu People’s Hospital, Yangzhou, 225001 Jiangsu China; 8 Department of Human Nutrition, University of Otago, Dunedin, 9016 New Zealand; 9 Gut Research Group, Faculty of Medicine, Universiti Kebangsaan Malaysia, 56000 Kuala Lumpur, Malaysia; 10 St George and Sutherland Clinical School, University of New South Wales, Sydney, NSW 2217 Australia

**Keywords:** Uric acid, Hyperuricemia, Gout, China, Urbanisation

## Abstract

**Background:**

Fructose plays an important role in the complex metabolism of uric acid in the human body. However, high blood uric acid concentration, known as hyperuricemia, is the main risk factor for development of gout. Therefore, we conducted an updated meta-analysis on the prevalence and geographical distribution of hyperuricemia among the general population in mainland China using systematic literature search.

**Methods:**

Five electronic databases were used to search for relevant articles published until 2019. All calculations were conducted using the Comprehensive Meta-Analysis (CMA) software. We included 108 eligible articles (172 studies by sex, 95 studies by regions, and 107 studies by study type) and an overall sample size of > 808,505 participants.

**Results:**

The pooled prevalence of hyperuricemia among the general population in mainland China was 17.4% (95% CI: 15.8–19.1%). Our subgroup analysis indicated that the pooled prevalence by regions ranged from 15.5 to 24.6%. Those living Northeast region and being males had the highest prevalence (*P* < 0.001). In addition, some provinces in South Central, East and Northeast regions reported a high prevalence (> 20%), particularly in males. An increasing prevalence was reported since 2005–2009 until 2015–2019. No publication of bias was observed as indicated by a symmetrical funnel plot and Begg and Mazumdar rank correlation (*P* = 0.392).

**Conclusion:**

Prevalence of hyperuricemia is increasing in China, and future studies should investigate the association between the prevalence of hyperuricemia and its risk factors in order to tackle the issue, particularly among the vulnerable groups. Also, our study was the first comprehensive study to investigate the overall prevalence of hyperuricemia in mainland China covering the six different regions.

## Background

High blood uric acid concentration, known as hyperuricemia, is the main risk factor for development of gout [1, 2]. Uric acid is a terminal metabolite of human purine compounds, which is slightly soluble in water and easy to form crystals [3, 4]. When uric acid increases to a certain threshold level in the human body, it is considered hyperuricemia [5].

The body has ~ 1200 mg and ~ 600 mg total body pool of exchangeable uric acid in males and females, respectively [6]. There are about 600 mg uric acid that are produced every day, and another 600 mg uric acid are excreted, resulting in a balanced state [7]. A disturbed state of purine metabolism can cause a variety of disorders, such as hyperuricemia, chronic gout, joint deformation and renal failure [3]. Among them, hyperuricemia has received increasing attention in recent decades because of its increasing global trends and risk of associated metabolic diseases. The prevalence of hyperuricemia can be influenced by several factors, including genetics, gender, age, lifestyle, diet, medication and economic development. For example, a higher prevalence is usually reported in the economically developed regions [8].

In addition, higher uric acid concentration is associated with increased risk of hospitalization, chronic kidney disease and cardiovascular disease (CVD), which can result in higher total medical costs and hospitalisation costs per patient. For example, the mean annual healthcare costs in Italy for hyperuricemic patients ranged from €2752 to €4607 [5]. Elderly patients with hyperuricemia in China are at risk of gout attacks caused by iatric problems, which may bring about complications such as deep vein thrombosis (DVT) and a prolonged hospital stay [9]. Therefore, this does not only increase the cost of medical treatment for patients, but also increase the cost of treatment for hospitals.

There are many observational studies on the prevalence of hyperuricemia, however most of them were focused on specific populations such as children from a region of mainland China. In addition, there are only two meta-analyses in the past that have examined the prevalence of hyperuricemia in mainland China; both with limitations [10, 11]. The first meta-analysis was conducted in 2011 with 59 articles [10] and the second one was in 2015 with 44 articles [11]; both did not have comprehensive coverage of the whole of China (for example, the former one did not include Inner Mongolia, while the latter one did not include Ningxia and Qinghai). Since China is the world’s most populous country with about 1.4 billion (i.e. 18.4% of the world population), updating the epidemiology of hyperuricemia can help to fill the gap in public health research and policy. To date, there have been no published English articles that have extensively reviewed the prevalence of hyperuricemia in mainland China until December 2019. Therefore, the aim of our study was to conduct a comprehensive review and quantitative meta-analysis on the prevalence of hyperuricemia in mainland China over the past two decades. In addition, analyses were also performed to provide a more detailed and updated epidemiological distribution of hyperuricemia by comparing different regions in mainland China.

## Methods

### Search strategy

**Fig. 1:**
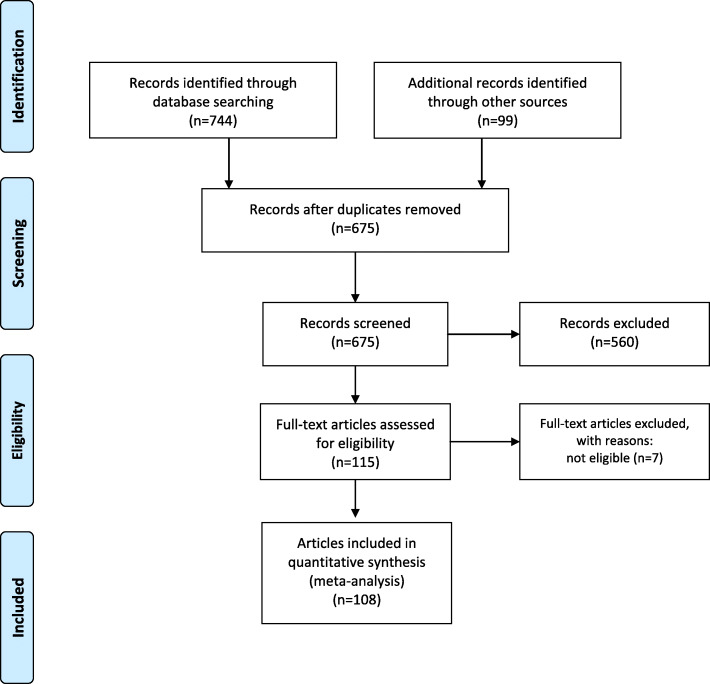
PRISMA flow diagram of the literature screening process

A systematic literature search from January 1995 to December 2019 was conducted for articles published in Chinese language from the following electronic databases: Wanfang Data, Shanghai Science and Technology Innovation Resources Center (SSTIR), China National Knowledge Infrastructure (CNKI) and Chinese Scientific Journals Fulltext Database (CQVIP). Keywords used in the database search included: “hyperuricemia” OR “high uric acid” OR “uric acid” OR “gout” AND “Chinese” OR “China” OR the name of the provinces in China. Database search results were entered into EndNote X8.2 file (Clarivate Analytics, New York, USA). The current systematic review and meta-analysis was conducted according to the Preferred Reporting Items for Systematic Reviews and Meta-Analyses (PRISMA) guidelines [12] (Fig. 1). The protocol of the systematic review and meta-analysis was registered at PROSPERO, as CRD42019141243, which is an international database of prospectively registered systematic reviews in health and social care. Since our systematic review and meta-analysis used data from published articles, there are no requirements for us to apply for the ethics approval. However, all human studies included in our systematic review and meta-analysis have been reviewed by the appropriate ethics committee in their institutions and have therefore been performed in accordance with the ethical standards laid down in an appropriate version of the WMA Declaration of Helsinki-Ethical Principles for Medical Research Involving Human Subject.

### Study selection

Studies were deemed to be eligible if they met the following criteria: (1) cross-sectional, cohort or case-control studies that were conducted in non-pregnant adults living in mainland China; (2) prevalence of hyperuricemia and sample size were reported; (3) detailed diagnostic criteria were included; and (4) full text of the article was able to be retrieved. Studies were excluded if they were: review articles and/or meta-analyses and inclusion of terminally ill or pregnant adults as participants.

### Quality assessment

The quality of eligible studies was independently assessed by two authors (J. H. and Z. F. M.) using a modified version of Newcastle-Ottawa Scale (NOS). When there were disagreements between the authors, they were resolved by discussion.

### Data extraction

For all eligible studies, the information about the authors, publication year, study design, age, sex, province, cases of hyperuricemia, total sample size, prevalence of hyperuricemia and cut-offs used for the determination of hyperuricemia was extracted. The corresponding authors of eligible studies were also contacted for obtaining the missing data in their articles.

### Statistical analysis

Meta-analysis was performed using the Comprehensive Meta-Analysis (CMA) software (V2.0, Biostat, Englewood, New Jersey). Random-effects models were used to estimate the pooled prevalence of hyperuricemia and 95% confidence intervals (CI) due to the large variation of study design among the included studies. Subgroup analyses were performed by province, study design, sex and study period. Heterogeneity tests were determined using the Q-test (*P* < 0.10) and I^2^ statistic (> 75%) [13]. Potential publication bias was assessed by the funnel plots and Begg and Mazumdar rank correlation (*P* < 0.05). The one-study-removed sensitivity analysis was performed to determine the possible causes of heterogeneity between the studies.

## Results

### Characteristics of the included studies

A total of 108 articles were identified after screening for relevancy and duplicates (Fig. 1). Table 1 shows a detailed description of the included studies in the systematic review and meta-analysis [10–12, 14–123]. All included studies were published between 1999 and 2019 and together comprised > 808,505 participants. Of the 108 articles, there were 172 studies by sex, 95 studies by regions, and 107 studies by study type (Table 2).

**Table 1:** Characteristics of the included studies in the systematic review and meta-analysis

No.	Study	Study type	Provinces (cities)/municipalities/autonomous regions	Region	Age (years)^**c**^	Case	Sample size	Prevalence (%)	Diagnostic cut-offs	Gender
1	Ma, Chen & Li (1999) [14]	CS	Guangdong	South Central	55–82	452	2041	22.1	>420 μmol/L	Both
						364	1696	21.5	>420 μmol/L	Male
						88	345	25.5	>420 μmol/L	Female
2	Shao et al. (2003) [15]	CS	Nanjing	East	≥18	1038	7778	13.3	NS	Both
						688	3790	17.6	≥417 μmol/L	Male
						370	3988	9.3	≥357 μmol/L	Female
3	Chen et al. (2004) [16]	CC	Anhui	East	45 ± 12	105	430	24.4	NS	Both
						70	227	30.8	>420 μmol/L	Male
						35	203	17.2	>360 μmol/L	Female
4	Wu et al. (2005) [17]	CS	Guangzhou, Guangdong	South Central	> 55	197	642	30.7	NS	Both
						46	152	30.3	>420 μmol/L	Male
						151	490	30.8	>350 μmol/L	Female
5	Yang et al. (2005) [18]	CS	Shandong	East	18–54	537	8640	6.2	NS	Both
						459	6289	7.3	≥416 μmol/L	Male
						78	2351	3.3	≥357 μmol/L	Female
6	Wang et al. (2006) [19]	CS	Shandong	East	20–80	269	2605	10.3	> 350 μmol/L	Female
7	Li et al. (2008) [20]	CH	China^a^	NA^a^	45–54	10	274	3.6	NS	Both
						5	90	5.6	≥416 μmol/L	Male
						5	184	2.7	≥356 μmol/L	Female
					55–64	18	307	5.9	NS	Both
						13	138	9.4	≥416 μmol/L	Male
						5	169	3.0	≥356 μmol/L	Female
					65–74	21	229	9.2	NS	Both
						12	116	10.3	≥416 μmol/L	Male
						9	113	8.0	≥356 μmol/L	Female
8	Fan et al. (2009) [118]	CS	Xinyang, Henan	South Central	40–75	738	5235	14.1	NS	Both
						379	1763	21.5	≥420 μmol/L	Male
						354	3472	10.2	≥360 μmol/L	Female
9	Lu et al. (2010) [21]	CS	Tianjin	North	22–53	19	151	12.6	≥410 μmol/L	Male
10	Yu et al. (2010) [22]	CS	Foshan, Guangdong	South Central	20–88	1117	7403	15.1	NS	Both
						714	3581	19.9	≥417 μmol/L	Male
						403	3822	10.5	≥357 μmol/L	Female
11	Yuan et al. (2011) [23]	CS	Guiyang	Southwest	> 60	399	2600	15.3	≥420 μmol/L	Both
						227	1430	15.9	NS	Male
						172	1170	14.7	NS	Female
12	Zhang & Zhang (2011) [24]	CS	China^a^	NA^a^	≥18	427	5774	7.4	NS	Both
13	Guo et al. (2012) [25]	CS	Taiyuan, Shanxi	Northwest	23–87	371	4228	8.8	NS	Both
						249	1308	19.0	≥420 μmol/L	Male
						122	2920	4.2	≥420 μmol/L	Female
14	Wang et al. (2012) [26]	CS	Yinchuan, Ningxia	Northwest	≥18	926	5921	15.6	NS	Both
						1352	7322	18.5	NS	Both
						1635	8717	18.8	NS	Both
15	Chen et al. (2013) [27]	CS	Guangxi	South Central	≥18	319	927	34.4	NS	Both
						157	419	30.9	NS	Male
						162	508	38.7	NS	Female
16	Duan et al. (2013) [28]	CS	Xinjiang	Northwest	≥18	261	2046	12.8	NS	Both
						228	823	27.7	>417 μmol/L	Male
						33	1223	2.7	>357 μmol/L	Female
17	Li et al. (2013) [29]	CS	Quanzhou, Fujian	East	40–80	253	1358	18.6	NS	Both
						99	363	27.3	≥416 μmol/L	Male
						154	995	15.5	≥357 μmol/L	Female
18	Li & Cao (2013) [30]	CS	Karamay, Xinjiang	Northwest	≥18	310	2032	15.3	NS	Both
						268	1086	24.7	NS	Male
						42	946	4.4	NS	Female
19	Lv et al. (2013) [31]	CS	Yantai, Shandong	East	31–78	66	635	10.4	≥380 μmol/L	Both
20	Su et al. (2013) [32]	CS	Nanhai, Guangdong	South Central	45–80	415	2015	20.6	NS	Both
						271	1110	24.4	>420 μmol/L	Male
						144	905	16.9	>357 μmol/L	Female
21	Wang et al. (2013) [33]	CS	Shanghai	East	40–70	58	1928	3.0	NS	Both
						33	582	5.7	>420 μmol/L	Male
						25	1346	1.9	>357 μmol/L	Female
22	Zhang, Wu & Lv (2013) [34]	CS	Hebei	North	21–95	693	3232	21.4	NS	Both
						446	1897	23.5	≥428 μmol/L	Male
						247	1335	18.5	≥357 μmol/L	Female
23	Zhou & He (2013) [35]	CH	Shenyang, Liaoning	Northeast	50–70	8	70	34.8	NS	Both
24	Chen, Dai & Lin (2014) [36]	CS	Guangzhou, Guangdong	South Central	45–75	603	1176	51.3	NS	Both
						341	612	55.7	>420 μmol/L	Male
						262	564	46.5	>357 μmol/L	Female
25	Cui et al. (2014) [37]	CS	Hebei	North	≥20	1091	7083	15.4	NS	Both
						904	5357	16.9	≥417 μmol/L	Male
						187	1726	10.8	≥357 μmol/L	Female
26	Li, Zhao, Gao (2014) [38]	CS	Yunnan	Southwest	27–89	367	2947	12.5	NS	Both
						303	1827	16.6	>420 μmol/L	Male
						64	1120	5.7	>360 μmol/L	Female
27	Lin et al. (2014) [39]	CS	Guangdong	South Central	> 60	190	1036	18.3	NS	Both
						86	383	22.5	≥420 μmol/L	Male
						104	653	15.9	≥420 μmol/L	Female
28	Liu et al. (2014) [40]	CS	Jilin	Northeast	38 ± 10	3395	16,807	20.2	NS	Both
						2930	9736	30.1	NS	Male
						465	7071	6.6	NS	Female
29	Pan et al. (2014) [41]	CS	Jiangsu	East	35–70	573	3122	18.4	NS	Both
						362	1349	26.8	≥420 μmol/L	Male
						211	1773	11.9	≥380 μmol/L	Female
30	Song et al. (2014) [42]	CS	Jiangxi	East	> 40	795	3795	20.9	NS	Both
						488	1824	26.8	>420 μmol/L	Male
						307	1971	15.6	>350 μmol/L	Female
31	Yong & Ye (2014) [43]	CS	Hebei	North	≥18–20	813	5269	15.4	NS	Both
						769	2717	28.3	>420 μmol/L	Male
						44	2552	1.7	>350 μmol/L	Female
32	Zhu, Wang, Liu (2014) [44]	CS	Xinjiang	Northwest	20–93	1489	10,025	14.9	NS	Both
33	Cao, Li & Yi (2015) [45]	CS	Guangzhou, Guangdong	South Central	20–80	290	988	29.4	NS	Both
						264	601	43.9	>420 μmol/L	Male
						26	387	6.7	>350 μmol/L	Female
34	Li et al. (2015a) [46]	CS	Gansu	Northwest	48 ± 15	392	2364	16.6	NS	Both
						256	1254	20.4	>420 μmol/L	Male
						136	1110	12.3	>360 μmol/L	Female
35	Li et al. (2015b) [47]	CS	Guangxi	South Central	≥20	14,181	51,206	27.7	NS	Both
						10,722	27,144	39.5	≥417 μmol/L	Male
						3459	24,062	14.4	≥357 μmol/L	Female
36	Li et al. (2015c) [48]	CS	Dongguan, Guangdong	South Central	≥18	519	1375	37.6	NS	Both
						366	657	26.6	>420 μmol/L	Male
						153	718	11.1	>350 μmol/L	Female
37	Liu et al. (2015) [11]	CS	Guangzhou, Guangdong	South Central	≥18	1334	4237	31.5	NS	Both
						859	2257	38.1	>420 μmol/L	Male
						475	1980	24.0	>360 μmol/L	Female
38	Lu (2015) [49]	CS	Shanghai	East	65–85	220	1128	19.5	NS	Both
						165	607	27.2	>420 μmol/L	Male
						63	511	12.3	>350 μmol/L	Female
39	Zhao (2015) [50]	CS	China^a^	NA^a^	20–60	4616	12,650	36.5	NS	Both
40	Zhou et al. (2015a) [51]	CS	Sichuan	Southwest	≥18	182	972	18.7	NS	Both
						123	452	27.2	≥420 μmol/L	Male
						59	520	11.3	≥360 μmol/L	Female
41	Zhou et al. (2015b) [52]	CS	Henan	South Central	20–60	1196	4916	24.3	NS	Both
						1128	4290	26.3	≥420 μmol/L	Male
						68	626	10.9	≥357 μmol/L	Female
42	Guli, He & Zhang (2016) [53]	CS	Gansu	Northwest	20–80	780	6400	12.2	>420 μmol/L	Both
43	Chen & Xing (2016) [54]	CS	Beijing	North	25–82	151	868	17.4	≥416 μmol/L	Male
44	Chen & Zhou (2016) [55]	CS	Zhejiang	East	> 60	691	4160	16.6	NS	Both
						393	2182	18.0	>420 μmol/L	Male
						298	1978	15.1	>360 μmol/L	Female
45	Fan et al. (2016) [56]	CS	Shanghai	East	≥18	5413	27,615	19.6	NS	Both
						3993	14,104	28.3	>420 μmol/L	Male
						1420	13,511	10.5	>357 μmol/L	Female
46	Feng et al. (2016) [57]	CS	Jiangsu	East	18–93	219	1352	16.2	NS	Both
						129	609	21.2	>420 μmol/L	Male
						90	743	12.1	>350 μmol/L	Female
47	Li (2016) [58]	CS	Tianjin	North	≥18	10,344	77,787	13.3	NS	Both
48	Li et al. (2016) [59]	CS	Chongqing	Southwest	39	1596	26,067	6.1	NS	Both
						1272	18,139	7.0	≥420 μmol/L	Male
						324	7928	4.1	≥357 μmol/L	Female
49	Liu et al. (2016) [60]	CS	Shanghai	East	≥18	8100	9653	83.9	NS	Both
						2872	3550	81.2	>420 μmol/L	Male
						5228	6103	85.9	>357 μmol/L	Female
50	Liu, Zhou & Yin (2016) [61]	CS	Yunnan	Southwest	32–60	131	390	33.6	NS	Both
						126	334	37.7	>420 μmol/L	Male
						5	56	9.1	>360 μmol/L	Female
51	Lu (2016) [62]	CS	Xinjiang	Northwest	≥60	233	986	23.6	NS	Both
52	Pu et al. (2016) [63]	CS	China^a^	NA^a^	20–91	1078	11,967	9.0	NS	Both
53	Wang (2016) [64]	CS	Hubei	South Central	18–22	358	4333	8.3	NS	Both
						294	2029	14.5	>420 μmol/L	Male
						64	2304	2.8	>350 μmol/L	Female
54	Xie et al. (2016) [65]	CS	Beijing; Tangshan and Zhangjiakou, Hebei	North	18–60	632	2782	22.7	NS	Both
						268	1830	14.6	>420 μmol/L	Male
						364	952	35.1	>357 μmol/L	Female
55	Yang, Wang & Wang (2016) [66]	CS	Tianjin	North	18–93	1165	8968	13.0	NS	Both
						959	5449	17.6	>417 μmol/L	Male
						206	3519	5.9	>357 μmol/L	Female
56	Zhang (2016) [67]	CS	China^a^	NA^a^	≥18	198	794	24.9	>420 μmol/L	Male
			Eastern China^a^	East	≥18	58	202	31.3	>421 μmol/L	Male
57	Zhao et al. (2016a) [68]	CS	Lanzhou, Gansu	Northwest	≥45	37	175	21.1	NS	Both
58	Zhao et al. (2016b) [69]	CS	Beijing	North	20 ± 3	1716	6400	26.8	NS	Both
						1464	4198	34.9	>417 μmol/L	Male
						252	2202	11.4	>357 μmol/L	Female
59	Zhao et al. (2016c) [70]	CS	Beijing	North	20–89	1086	6690	16.2	NS	Both
						785	3339	23.5	>417 μmol/L	Male
						301	3351	10.0	>357 μmol/L	Female
60	Feng et al. (2017) [71]	CS	Beijing	North	range ≥ 18	2257	12,335	18.3	NS	Both
						1867	7681	24.3	>420 μmol/L	Male
						390	4654	8.4	>357 μmol/L	Female
61	Guo et al. (2017) [72]	CS	Heilongjiang	Northeast	20–59	419	1477	28.4	>420 μmol/L	Male
62	He (2017) [73]	CS	Dalian, Liaoning	Northeast	22–91	358	2002	17.9	NS	Both
						252	1044	24.1	>420 μmol/L	Male
						106	958	11.1	premenopausal>350 μmol/L postmenopausal>420 μmol/L	Female
63	Li et al. (2017) [74]	CC	Urumqi, Xinjiang	Northwest	18–78	221	1644	23.8	NS	Both
64	Li, Zhou & Pan (2017) [75]	CS	Guangdong	South Central	22–90	314	3071	10.2	NS	Both
65	Lin et al. (2017) [76]	CS	Yunnan	South Central	18–84	196	1682	11.7	NS	Both
						139	923	15.1	≥417 μmol/L	Male
						57	759	7.5	≥357 μmol/L	Female
66	Liu et al. (2017a) [77]	CS	Shanghai	East	≥18	148	908	16.3	NS	Both
						48	308	15.6	>420 μmol/L	Male
						100	600	16.7	>360 μmol/L	Female
67	Liu et al. (2017b) [78]	CS	Shanghai	East	20–80	1444	9294	15.5	NS	Both
						639	3393	18.8	>420 μmol/L	Male
						805	5901	13.6	>357 μmol/L	Female
68	Liu et al. (2017c) [79]	CS	Hunan	South Central	20–80	1435	5356	26.8	NS	Both
						1234	3489	35.4	NS	Male
						201	1867	10.8	NS	Female
69	Liu, Yan & Li (2017) [80]	CS	Hebei	North	≥18	698	6045	11.5	NS	Both
						488	3344	14.6	>416 μmol/L	Male
						210	2701	7.8	>357 μmol/L	Female
70	Liu & Yang (2017) [81]	CC	Beijing	North	21–67	204	1799	11.3	NS	Both
71	Min (2017)	CS	Shenyang, Liaoning	Northeast		74	282	26.2	NS	Both
72	Pan & Jiang (2017) [82]	CS	Fuzhou, Fujian	East	75	210	744	28.2	NS	Both
						196	618	31.7	>420 μmol/L	Male
						14	126	11.1	>420 μmol/L	Female
73	Wang & Bai (2017) [83]	CS	Ningxia	Northwest	22–60	121	1012	12.0	NS	Both
						99	757	13.1	>420 μmol/L	Male
						22	255	8.6	>357 μmol/L	Female
74	Wang & Bao (2017) [84]	CS	Shanghai	East	60–93	454	2426	18.7	NS	Both
						220	1076	20.5	>420 μmol/L	Male
						234	1350	17.3	>360 μmol/L	Female
75	Xie et al. (2017) [85]	CS	Guangdong	South Central	35–75	279	2587	10.8	NS	Both
						175	1410	12.4	>417 μmol/L	Male
						104	1177	8.8	>357 μmol/L	Female
76	Yu & Jie (2017) [86]	CS	Shandong	East	21–76	1191	10,743	11.1	NS	Both
						1116	6426	10.4	≥430 μmol/L	Male
						75	4317	0.7	≥375 μmol/L	Female
77	Zhang (2017a) [87]	CS	Liaoning	Northeast	21–50	121	500	24.2	NS	Both
78	Zhang (2017b) [88]	CS	Anhui	East	25–87	19	230	8.3	>420 μmol/L	Both
79	Zhang, Chen & Liu (2017) [89]	CS	Zhuhai, Guangdong	South Central	18–75	590	1834		NS	Both
						290	679	42.7	NS	Male
						300	1155	26.0	NS	Female
80	Zheng (2017) [90]	CS	China^a^	NA^a^	24 ± 6	432	1721	25.1	> 420 μmol/L	Male
81	Chen et al. (2018a) [91]	CS	Liaoning, Heilonjiang, Shandong, Henan, Hubei, Hunan, Jiangsu, Guizhou, Guangxi	NA^b^	49 ± 17	1435	8785	16.3	NS	Both
						886	4110	21.6	≥420 μmol/L	Male
						549	4675	11.7	≥360 μmol/L	Female
82	Chen et al. (2018b) [92]	CS	Guangxi	South Central	> 60	161	817	19.7	>420 μmol/L	Both
83	Chen et al. (2018c) [93]	CS	Guangdong	South Central	≥18	328	981	33.4	>420 μmol/L	Male
84	Chen et al. (2018d) [94]	CS	Guangxi	South Central	65–96	241	1223	19.7	NS	Both
						163	629	25.9	≥420 μmol/L	Male
						78	594	13.1	≥360 μmol/L	Female
85	Fan, Mao & Chen (2018) [95]	CS	Ningbo, Zhejiang	East	≥45	750	3395	22.1	NS	Both
86	He (2018) [96]	CS	Henan	South Central	25–89	410	2193	18.7	NS	Both
						305	1156	26.4	>420 μmol/L	Male
						105	1037	10.1	>350 μmol/L	Female
87	Hu et al. (2018) [97]	CS	Guangxi	South Central	20–70	1035	6241	16.6	NS	Both
						755	3271	23.1	> 420 μmol/L	Male
						280	2970	9.4	> 360 μmol/L	Female
88	Huang & Huang (2018) [98]	CS	Guangzhou, Guangdong	South Central	51–82	55	338	16.3	NS	Both
						49	289	17.0	NS	Male
						6	49	12.2	NS	Female
89	Huang et al. (2018) [99]	CS	Guizhou	Southwest	18–75	26,341	143,687	18.3	NS	Both
						15,387	75,364	20.4	≥417 μmol/L	Male
						20,954	68,323	16.0	≥357 μmol/L	Female
90	Li, Wang & Xu (2018) [100]	CS	Beijing	North	18–80	255	1700	15.0	NS	Both
						116	620	18.7	NS	Male
						139	1080	12.9	NS	Female
91	Lin et al. (2018a) [101]	CS	Fujian	East	18–63	666	2666	25.0	NS	Both
						411	1251	43.9	>417 μmol/L	Male
						255	1415	18.0	>357 μmol/L	Female
92	Lin et al. (2018b) [102]	CS	Guangzhou, Guangdong	South Central	≥18	1642	5603	29.3	NS	Both
						1590	5281	30.1	>420 μmol/L	Male
						53	322	16.5	>350 μmol/L	Female
93	Lu (2018a) [103]	CS	Zhejiang	East	55	147	1200	12.3	NS	Both
						93	597	15.6	> 420 μmol/L	Male
						54	603	9.0	> 350 μmol/L	Female
94	Lu (2018b) [104]	CH	Inner Mongolia	North	≥35	383	2554	15.0	NS	Both
						331	1632	20.3	>420 μmol/L	Male
						52	922	5.6	>360 μmol/L	Female
						477	2554	18.7	NS	Both
						413	1632	25.3	>420 μmol/L	Male
						64	922	6.9	>360 μmol/L	Female
						511	2554	20.0	NS	Both
						446	1632	27.3	>420 μmol/L	Male
						65	922	7.6	>360 μmol/L	Female
						530	2554	20.8	NS	Both
						465	1632	28.5	>420 μmol/L	Male
						65	922	8.0	>360 μmol/L	Female
95	Su et al. (2018) [105]	CS	Zhejiang	East	range ≥ 18	694	3905	17.8	NS	Both
						364	1797	20.3	NS	Male
						330	2108	15.7	NS	Female
96	Tuo et al. (2018) [106]	CS	Gansu	Northwest	20–80	768	4263	18.0	NS	Both
						432	1783	24.2	≥420 μmol/L	Male
						336	2480	13.6	≥350 μmol/L	Female
97	Wang et al. (2018a) [107]	CS	Beijing; Xi’an, Shaanxi; Harbin, Heilongjiang; Chengdu, Sichuan; Chongqing; Changsha, Hunan; Shanghai	NA^b^	≥60	754	5351	14.1	NS	Both
						304	2304	13.2	≥420 μmol/L	Male
						450	3047	14.8	≥360 μmol/L	Female
98	Wang et al. (2018b) [108]	CS	Liaoning; Heilongjiang; Jiangsu; Shandong; Henan; Hubei; Hunan; Guangxi	NA^b^	≥18	555	4111	13.5	NS	Both
						361	1871	19.3	> 418 μmol/L	Male
						194	2240	8.7	> 357 μmol/L	Female
99	Wang & Ma (2018) [109]	CS	Liaoning	Northeast	22–65	432	1481	29.2	> 420 μmol/L	Male
100	Yang et al. (2018) [110]	CS	China^a^	NA^a^	≥18	3855	24,095	16.0	NS	Both
101	Yu et al. (2018) [111]	CS	Xinjiang	Northwest	30–81	2648	14,426	18.4	NS	Both
102	Zhang et al. (2018) [112]	CS	Ningxia	Northwest	≥18	3880	19,356	20.0	NS	Both
						3180	12,115	26.2	>420 μmol/L	Male
						700	7241	9.7	>350 μmol/L	Female
103	Zhou et al. (2018) [113]	CS	Ningxia	Northwest	≥35	279	1743	16.0	NS	Both
						193	1044	18.5	NS	Male
						86	699	12.3	NS	Female
104	Hu, Zhao & Shang (2019) [114]	CS	Tibet	Northwest	20–49	170	1669	10.2	NS	Both
						114	952	12.0	NS	Male
						56	717	7.8	NS	Female
105	Tian et al. (2019) [115]	CS	Beijing	North	18–97	10,795	52,673	20.5	NS	Both
						8524	27,419	31.1	NS	Male
						2271	25,254	9.0	NS	Female
106	Wang et al. (2019) [123]	CC	China^a^	NA^a^	≥18	2977	22,983	13.0	NS	Both
						1999	10,787	18.5	NS	Male
						978	12,796	7.6	NS	Female
107	Yang (2019) [116]	CH	Guilin, Guangxi	South Central	20–68	160	1545	10.4	NS	Both
108	Yu et al. (2019) [117]	CS	Shenyang, Liaoning	Northeast	≥18	7705	14,323	53.7	NS	Both

*CS* Cross-sectional, *CC* Case control, *CH* Cohort study, *NA* Not applicable, *NS* Not stated

^a^No specific provinces were reported

^b^More than one region was involved

^c^Mean used unless range reported

**Table 2:** Prevalence of hyperuricemia by subgroups in mainland China

Subgroups	No. of studies	Pooled	95% CI	I^**2**^ (%)	***P***-value
**Region**					
*East*	23	0.173	0.139–0.213	99.844	< 0.001
*North*	16	0.174	0.134–0.222	99.241	< 0.001
*Northeast*	6	0.246	0.163–0.353	99.873	< 0.001
*Northwest*	18	0.155	0.121–0.197	97.447	< 0.001
*South Central*	26	0.207	0.170–0.249	99.373	< 0.001
*Southwest*	6	0.158	0.102–0.236	99.779	< 0.001
Overall	95	0.181	0.163–0.201	99.734	0.281
**Sex**					
*Females*	83	0.110	0.096–0.126	99.678	< 0.001
*Males*	89	0.227	0.202–0.254	99.447	< 0.001
Overall	172	0.163	0.149–0.178	99.613	< 0.001
**Study type**					
*Cross-sectional*	94	0.181	0.164–0.200	99.761	< 0.001
*Cohort*	9	0.119	0.082–0.169	95.073	< 0.001
*Case control*	4	0.149	0.088–0.240	94.186	< 0.001
Overall	107	0.174	0.158–0.191	99.735	0.062

### Pooled prevalence of hyperuricemia

**Fig. 2:**
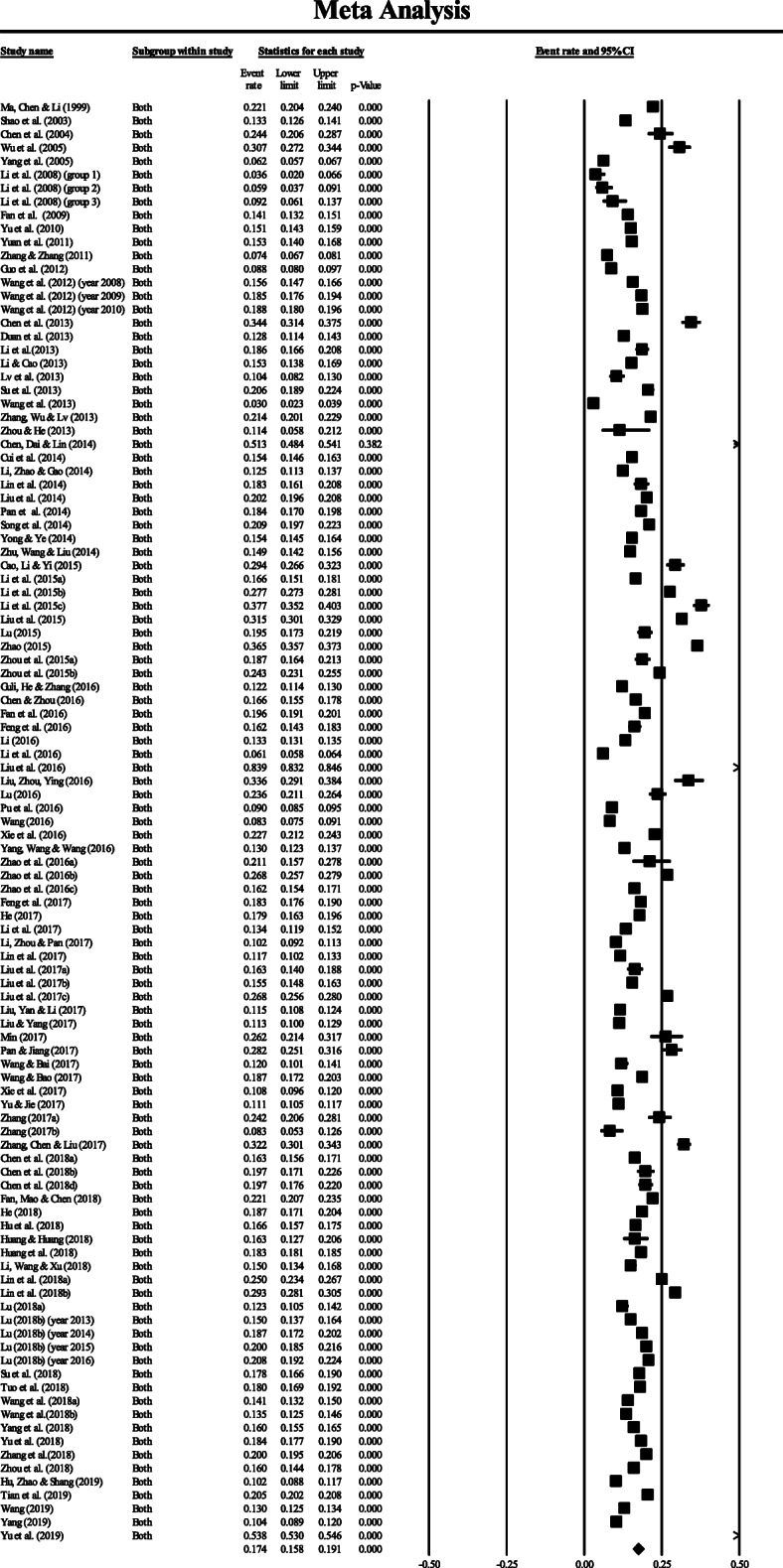
Forest plot of the pooled prevalence and 95% CI of hyperuricemia among the general population in mainland China

The pooled estimate of prevalence in the general population was 0.174 (95%CI: 0.158–0.191) (Fig. 2), which suggested that 17.4% of the population in mainland China had hyperuricemia.

### Subgroup analysis

The prevalence of hyperuricemia was analysed in subgroups, which were categorised according to the following categories: provinces/municipalities/autonomous regions, regions (northeast, northwest, north, southwest, south central and east), sex, study type and year.

The pooled prevalence of hyperuricemia by regions ranged from 15.5 to 24.6%. The pooled prevalence in Northeast region was the highest (24.6%), followed by South Central (20.7%), East (17.3%), North (17.4%), Southwest (15.8%), and Northwest (15.5%) (Table 2). In terms of gender distribution, the pooled prevalence of hyperuricemia in males was significantly higher than females (22.7% (95% CI: 20.2–25.4%) vs. 11.0% (95% CI: 9.6–12.6%)) (*P* < 0.001) (Table 2). For the study types, there was no difference in prevalence (*P* = 0.062) and the range of prevalence of hyperuricemia was from 11.9 to 18.1%.

**Fig. 3:**
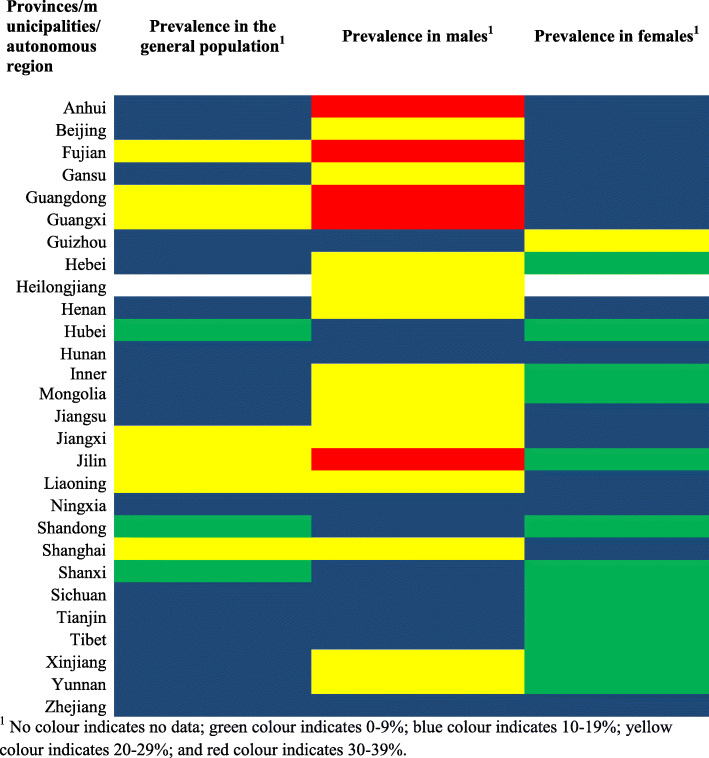
Prevalence of hyperuricemia in mainland China according to different provinces, municipalities and autonomous regions

Figure 3 shows the prevalence of hyperuricemia in mainland China by different provinces, municipalities and autonomous regions. Shanghai, Jiangxi, Jilin, Liaoning, Fujian, Guangdong and Guangxi reported a high prevalence of hyperuricemia ≥20%, while Hubei, Shandong and Shanxi had a low prevalence of hyperuricemia < 10%. The remaining provinces, municipalities and autonomous regions had a moderate prevalence of hyperuricemia (10–19%). For males, five provinces (i.e. Anhui, Guangdong, Guangxi, Jilin, and Fujian) reported a very high prevalence of hyperuricemia ≥30% and the remaining provinces, municipalities and autonomous regions reported a moderate-to-high prevalence of hyperuricemia ≥10–29%. For females, majority of the provinces, municipalities and autonomous regions reported a low-to-moderate prevalence of hyperuricemia (0–19%), while Guizhou was the only province with high prevalence of hyperuricemia (≥20%).

In the general population, there was a downward trend in the prevalence of hyperuricemia from 1995 to 1999 (22.1%) to 2015–2019 (18.6%). Similar downwards trends in the prevalence of hyperuricemia for males and females were also observed.

### Analysis of heterogeneity and publication bias

**Fig. 4:**
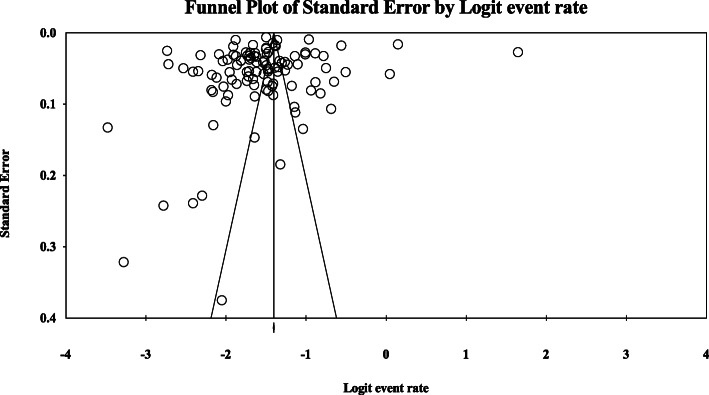
Funnel plot for the meta-analysis of the prevalence of hyperuricemia in mainland China

There was a significant heterogeneity in the included studies (I^2^ = 99.735%, *P* < 0.001). However, no indications of publication bias were observed as indicated by a symmetrical funnel plot (Fig. 4) and Begg and Mazumdar rank correlation (*P* = 0.392). The overall results remained unchanged as well after we performed a trim and fill method. Similarly, no publication bias was also reported for the subgroups analysis (Begg and Mazumdar rank correlation with a *P*-value > 0.05) and all funnel plots were symmetrical.

## Discussion

We performed a comprehensive meta-analysis of 108 observational studies over two decades and covered 27 provinces, autonomous regions and municipalities in the mainland China. In our meta-analysis, the prevalence of hyperuricemia in the general population of mainland China was 17.4% (22.7% in males and 11.0% in females), which was within the range of reported global prevalence (ranging from 1 to 85%) [8].

Our pooled prevalence was higher than a meta-analysis reported by Liu et al. i.e. 13.3% (19.4% in males and 7.9% in females) [11]. Our prevalence was similar to some developing countries in Asia. In Thailand, the overall prevalence of hyperuricemia was 10.6% in the general population with 18.4 and 7.8% in males and females, respectively [124]. In Turkey, the overall prevalence of hyperuricemia was 12.1% and males had a higher prevalence than females (i.e. 19.0% vs. 5.8%) [125].

However, our results were lower than that reported in developed countries [122, 126]. In the United States, the prevalence of hyperuricemia was 21.2 and 21.6% in males and females, respectively [126]. In Japan, the prevalence of hyperuricemia in the general population was 25.8% (34.5 and 11.6% in males and females, respectively) [122]. The higher prevalence reported in developed countries was most likely due to rapid aging and urbanisation [126]. In addition, the prevalence of non-communicable disease and obesity has also increased in these developed countries [122, 126], which might have contributed to the higher prevalence of hyperuricemia. Therefore, we strongly recommend that the Chinese health authorities should introduce more effective public health policies measures including prevention of obesity programme and promotion of health lifestyles to reduce the prevalence of hyperuricemia in Chinese population.

Since China is a vast country characterised by distinct regions, the prevalence of hyperuricemia varies largely in different provinces and regions. Our results reported that the prevalence of hyperuricemia ranged from 15.8 to 24.6%, with the highest prevalence in the Northeast region. We postulated that the large variability in the prevalence might be caused by the difference in the economic development and sedentary lifestyle adopted in these regions and provinces. For example, those living in Guangxi, Guangdong, Fujian and Jiangxi, people would consume more meat, alcohol and seafood. These foods are rich in purine which can cause an increase in the production of uric acid in the body [127]. Shanghai is one of the most economically developed areas in China. Rapid economic growth has led to unhealthy lifestyles and dietary patterns in the Shanghai population. In addition, an increased inactivity at work has also contributed to a higher prevalence of hyperuricemia [128]. In Jilin and Liaoning, we also reported a high prevalence of hyperruricemia (20–29%), which could be due to the high consumption of alcohol intake, particularly beer and liquor [129]. However, the specific reasons why these regions had a high prevalence require further research. In addition, with these results, the management of hyperuricemia (including routine health check-ups and serum uric acid screening tests) in these regions can be better implemented and improved by the health authorities. Nutrition education and lifestyle interventions can also be developed and specifically targeted to the high risk regions with proper healthcare resources by the health authorities. This is because if hyperuricemia is not well managed and prevented especially in regions with high prevalence, it can induce several medical complication including chronic failure and gout, which increases the cost of medical care [2].

In addition, we reported that males had a significantly higher prevalence of hyperuricemia than females (22.7% vs. 11.0%). Such a difference might be due to the sex hormones [130]. Serum uric acid level is generally higher in males than females. This is because there is an increase renal urate clearance by estrogen in women [129]. Our findings were consistent with the results reported in several countries from Asia and the Asia Pacific region including Nepal [131], Thailand [132], Turkey [125], Saudi Arabia [133], Seychelles [134], Japan [122] and New Zealand [135].

Our study also reported an increasing prevalence of hyperuricemia over time in males and females. We speculated that factors including aging population and obesity have contributed to the increase [126]. However, we also noticed different diagnostic cut-offs were used to diagnose hyperuricemia. It will be helpful to compare these different cut-offs in the same population in order to understand their validity in diagnosing hyperuricemia.

Our meta-analysis has several strengths. Firstly, to our knowledge, our study is the most comprehensive study among the general population in mainland China. Unlike the previous two meta-analyses [10, 11], our sample size (> 808,505 participants) and number of eligible articles (*n* = 108) were larger; and we included analyses on differences across regions, provinces, sex and study periods. Secondly, our pooled data covered all the six regions in China. In addition, all the provinces, municipalities and autonomous regions were also included, except for Qinghai, Chongqing, Hong Kong, Macao and Hainan. Thirdly, the authors who were involved in the data extraction and interpretation were proficient in the Chinese language. However, our study also suffered from a few limitations. Most of the included articles were cross-sectional studies. Since the definition of hyperuricemia varied according to the diagnostic cut-offs used by different studies, this factor should also be taken into consideration when interpreting these results. There was also a large heterogeneity in the quality of the articles, although no indications of publication bias were reported. We also did not make a clear distinction between urban and rural areas. Therefore, future studies with larger populations should consider investigate if health literacy, health status, sociodemographics and physical activity level play an important factor in the prevention and management of hyperuricemia, especially in adolescents, pregnant women and older adults with lower socioeconomic status [136].

## Conclusions

Hyperuricemia has become an important public health problem in mainland China, particularly among males. Special attention should be paid to the residents in geographical regions with high prevalence of hyperuricemia. In addition, our study was the first comprehensive study to investigate the overall prevalence of hyperuricemia in mainland China covering the six regions. Our study also underline the importance of having more larger population-based intervention studies to tackle the increasing problem of hyperuricemia, particularly the vulnerable groups in mainland China. Future studies should investigate the association between the prevalence of hyperuricemia and its risk factors such as geographical region, economic level and sex in order to develop public health policies for tackling the issue.

## Data Availability

Not applicable.

## Abbreviations

CMA: Comprehensive Meta-Analysis
CVD: Cardiovascular disease
SSTIR: Shanghai Science and Technology Innovation Resources Center
CNKI: China National Knowledge Infrastructure
CQVIP: Chinese Scientific Journals Fulltext Database
PRISMA: Preferred Reporting Items for Systematic Reviews and Meta-Analyses
NOS: Newcastle-Ottawa Scale
CI: Confidence intervals
